# Phylogenomic analysis of the chloroplast genome of the green-tide forming macroalga *Ulva intestinalis* Linnaeus (Ulvophyceae, Chlorophyta)

**DOI:** 10.1080/23802359.2021.1978889

**Published:** 2021-09-24

**Authors:** Hongshu Wang, Feng Liu, Jing Wang, Nansheng Chen

**Affiliations:** a National Engineering Research Center for Marine Aquaculture, Zhejiang Ocean University, Zhoushan, Zhejiang, China; b CAS Key Laboratory of Marine Ecology and Environmental Sciences (KLMEES), Institute of Oceanology, Chinese Academy of Sciences (IOCAS), Qingdao, Shandong, China; c Marine Ecology and Environmental Science Laboratory, Pilot National Laboratory for Marine Science and Technology (Qingdao), Qingdao, Shandong, China; d Center for Ocean Mega-Science, Chinese Academy of Sciences, Qingdao, Shandong, China

**Keywords:** *Ulva intestinalis*, chloroplast genome, phylogenomic analysis, Ulvophyceae, group I/II intron

## Abstract

*Ulva intestinalis* Linnaeus 1753 (Ulvophyceae, Chlorophyta) is a marine green macroalga that is distributed on coasts of the Yellow Sea and the Bohai Sea in China. Here, the complete chloroplast genome of *U. intestinalis* was constructed and analyzed comparatively. The chloroplast genome of *U. intestinalis* is a 99,041-bp circular molecule that harbors a total of 112 genes including 71 protein-coding genes (PCGs), 26 transfer RNA genes (tRNAs), three ribosomal RNA genes (rRNAs), three free-standing open reading frames (*orf*s) and nine intronic *orf*s, and ten introns in seven genes (*atpA*, *infA*, *psbB*, *psbC*, *petB*, *rrnL*, and *rrnS*). The maximum likelihood (ML) phylogenomic analysis shows that *U. intestinalis* firstly groups with *Ulva compressa*, and then these two species together with the *Ulva australis*–*Ulva fenestrata*–*Ulva rotundata* subclade form a monophyletic clade, *Ulva* lineage II. *U. intestinalis* chloroplast genome is the only one in *Ulva* lineage II where the reversal of a collinear block of two genes (*psbD*–*psbC*) did not occur, and its genome structure is consistent with that of most chloroplast genomes in *Ulva* lineage I, indicating that the similarity of genome structure is not completely related to the genetic relationship of *Ulva* species. Our genomic data will facilitate the development of specific high-resolution chloroplast molecular markers for rapid identification of *U. intestinalis*, and help us understand its population diversity and genetic characteristics on a global scale.

The macroalgal genus *Ulva* Linnaeus 1753 (Ulvophyceae, Chlorophyta) includes ∼85 species of green seaweeds that grow in marine, estuarine and freshwater environments worldwide (Guiry and Guiry 2021). It is noticeable that *Ulva* species are opportunistic algae that tend to form green tides and marine fouling in nutrient-rich waters around the world (Liu et al. 2013; Wang et al. 2015). Thus far, the complete chloroplast genomes (cpDNAs) have been sequenced and constructed for 14 species in genus *Ulva* (e.g. Melton et al. 2015; Cai et al. 2017; Suzuki et al. 2018; Fort et al. 2021), and these *Ulva* cpDNAs share the same set of 100 conserved genes including 71 protein-coding genes (PCGs), 26 transfer RNA genes (tRNAs), three ribosomal RNA genes (rRNAs), and show different genome architectures due to multiple rearrangement events (Liu and Melton 2021).

*Ulva intestinalis* Linnaeus 1753 is a common marine green seaweed that has a cosmopolitan distribution with cases reported from the coasts of Asia, Australia, Europe, and America (Hayden and Waaland 2004; Heesch et al. 2009; Kirkendale et al. 2013; Steinhagen et al. 2019), and is also well-known as a typical green-tide forming macroalga (Leskinen et al. 2004; Kostamo et al. 2008). In China, this alga is distributed from temperate coasts (e.g. Yellow Sea and Bohai Sea) to subtropical coasts (e.g. South China Sea) (Titlyanova et al. 2012). In this study, we collected the thalli of *U. intestinalis* from the intertidal zone of Zhifu District (N 37°32′21″, E 121°24′29″), Yantai, Shandong Province, China in November 2020. Its specimen was deposited in the collection of marine algae in KLMEES of IOCAS (Feng Liu, liufeng@qdio.ac.cn) under the voucher number 2020-YT-Uin-1. Fresh algal tissue from one individual thallus was used for DNA extraction using a Plant Genome DNA Kit (DP305, Tiangen Biotech, Beijing, China). The extracted DNA was segmented by Covaris S220 ultrasonic crater. The qualified libraries were sequenced on an Illumina NovaSeq platform (Illumina, USA) using paired-end sequencing. The complete chloroplast genome of *U. intestinalis* was constructed by the GetOrganelle software. The annotation of PCGs, tRNAs, rRNAs, open reading frames (*orf*s), and introns was performed using Open Reading Frame Finder (https://www.ncbi.nlm.nih.gov/orffinder), tRNAscan-SE 2.0 (Chan et al. 2021), and RNAweasel Tool (https://megasun.bch.umontreal.ca/cgi-bin/RNAweasel).

The chloroplast genome of *U. intestinalis* is a 99,041-bp circular molecule (GenBank accession number: MZ158703) with the G + C content of 24.97%. This genome harbored a total of 112 genes including 71 PCGs, 26 tRNAs, three rRNAs, three specific free-standing *orf*s, and nine intronic *orf*s (intron encoded proteins). Ten introns are identified to be present in seven genes including *atpA* (one intron), *infA* (one), *psbB* (three), *psbC* (one), *petB* (one), *rrnL* (two), and *rrnS* (one). Introns embedded in *atpA*, *psbB*, *psbC*, *rrnL*, and *rrnS* belong to group I intron, each of which contained an *orf* encoding a putative LAGLIDADG, or GIY-YIG, or H-N-H homing endonuclease. Introns embedded in *infA* and *petB* belong to group II intron (Liu and Melton 2021). The intron *infA*-62 in *U. intestinalis* is 596 bp in size and its intronic *orf* was completely degenerate. The intron *petB-69* is 2216 bp in size and harbors an intronic *orf* which encodes a putative reverse transcriptase/maturase (RTM). The three specific *orfs* including *orf103*, *orf136*, and *orf169* had little sequence similarity to any PCGs in the GenBank based on the search of blastp.

The phylogenomic tree is constructed with 1000 bootstrap replicates based on maximum likelihood (ML) analysis of amino acid (aa) sequences of 71 common PCGs from chloroplast genomes of 15 *Ulva* species, using MEGA 7.0. The initial tree for the heuristic search was obtained by applying the Neighbor-Joining method to a matrix of pairwise distances estimated using a JTT model (Kumar et al. 2016). There were a total of 22,776 positions in the final aa dataset. The phylogenomic analysis showed that 15 *Ulva* species fell into two clades, *Ulva* lineage I and II, with high bootstrap support values (100%). *U. intestinalis* firstly groups with *Ulva compressa*, and then these two species together with the *Ulva australis*–*Ulva fenestrata*–*Ulva rotundata* subclade forms a monophyletic clade, *Ulva* lineage II (Figure 1). Previously, we found that a locally collinear block of two genes (*psbD*–*psbC*) has been inverted in chloroplast genomes of *Ulva* lineage II including *U. compressa*, *U. australis*, *U. fenestrata*, and *U. rotundata* (Liu and Melton 2021), whereas this reversal event did not occur in *U. intestinalis* cpDNA. The genome structure of *U. intestinalis* cpDNA is consistent with that of most chloroplast genomes in *Ulva* lineage I, excluding *Ulva* sp. and *Ulva meridionalis*, indicating that the similarity of genome structure is not completely related to the genetic relationship of *Ulva* species.

**Figure 1.**
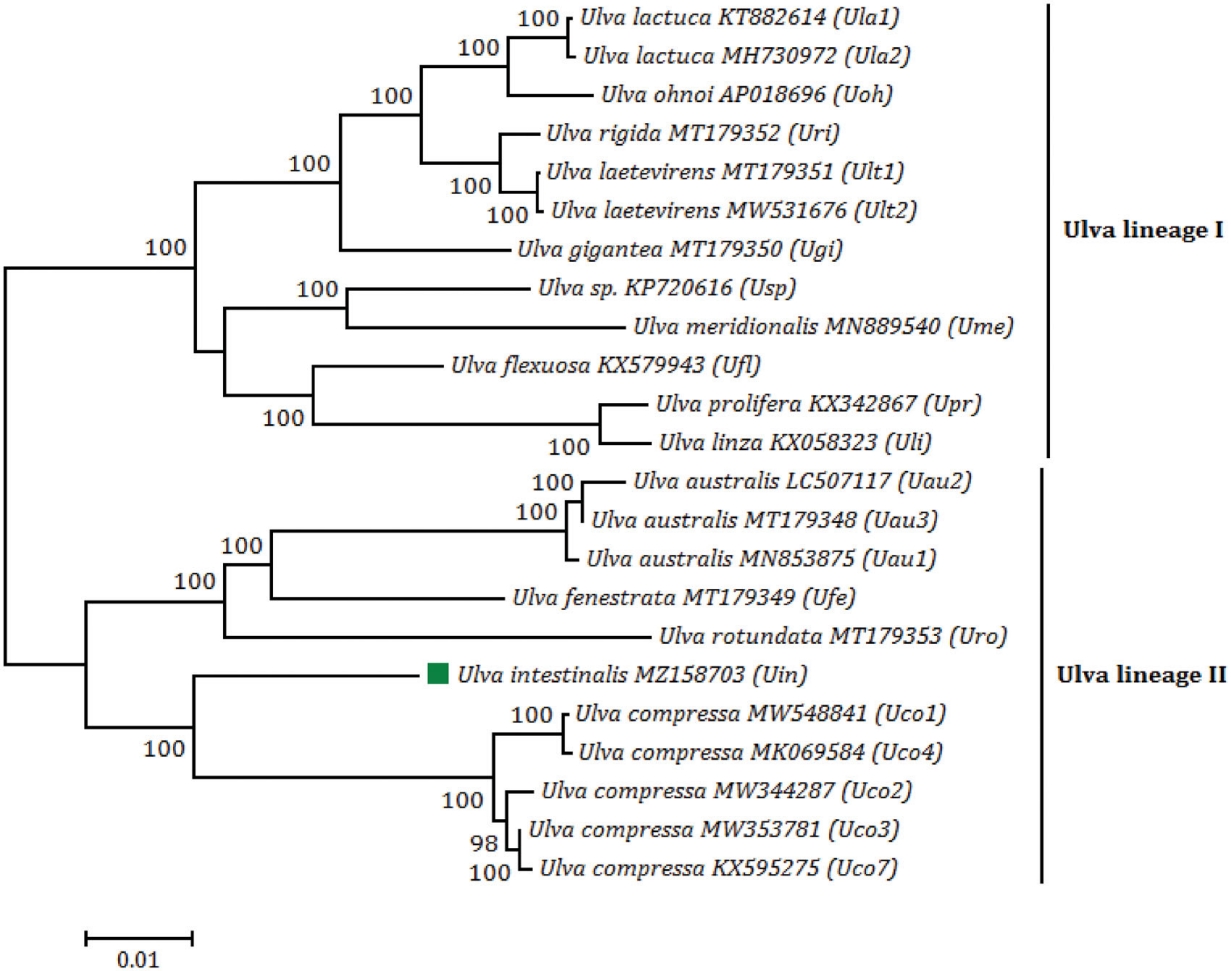
The maximum likelihood (ML) phylogenomic tree was constructed with 1000 bootstrap replicates based on amino acid (aa) sequences of 71 common PCGs from chloroplast genomes of 15 *Ulva* species, using MEGA 7.0. *U. compressa* MK069585 (*Uco5*) and *U. compressa* MT916929 (*Uco6*) were absent in this tree, due to their incomplete chloroplast genomes and faulty assembly of some PCGs (*rpoA*, *rpoB*, *rpoC1*, and *rpoC2*) (Liu and Melton 2021). Branch lengths are proportional to the amount of sequence change, which are indicated by the scale bar below the trees.

*U. intestinalis* displays great intraspecific morphological and cytological variation at different growth stages or under different environmental conditions (Leskinen et al. 2004), which makes it difficult to identify species solely based on morphological characteristics. Our genomic data in this study will facilitate the development of specific high-resolution chloroplast molecular markers for rapid identification of the green-tide forming alga, *U. intestinalis*, and help us understand its population diversity and genetic characteristics on a global scale.

## Data Availability

The genome sequence data that support the findings of this study are openly available in GenBank of NCBI under the accession number MZ158703 (https://www.ncbi.nlm.nih.gov/nuccore/MZ158703.1). The associated BioProject, SRA, and Bio-Sample numbers are PRJNA728799 (https://www.ncbi.nlm.nih.gov/bioproject/728799), SRR14493638 (https://trace.ncbi.nlm.nih.gov/Traces/sra/?run=SRR14493638), and SAMN19103542 (https://www.ncbi.nlm.nih.gov/biosample/SAMN19103542), respectively.
